# Clinical Characteristics of Patients With Chronic Stevens-Johnson Syndrome Treated at a Major Tertiary Eye Hospital Within the United Kingdom

**DOI:** 10.3389/fmed.2021.644795

**Published:** 2021-05-24

**Authors:** Samir Jabbour, Nizar Din, Abison Logeswaran, Sara Taberno Sanchez, Sajjad Ahmad

**Affiliations:** ^1^Moorfields Eye Hospital, London, United Kingdom; ^2^Department of Cornea & External Diseases, Institute of Ophthalmology, University College London, London, United Kingdom

**Keywords:** Stevens Johnson syndrome, toxic epidermal necrolysis, Limbal stem cell failure, ocular surface disease, symblepharon

## Abstract

The purpose of this study is to provide a comprehensive review of the clinical characteristics in chronic Stevens-Johnson syndrome (SJS) patients within the United Kingdom population, their causative factors, treatment profile and prognosis. This retrospective series included 91 patients with chronic SJS treated at Moorfields Eye Hospital (London, United Kingdom). A chart review included visual acuity and presence of clinical findings (including lid abnormalities and ocular surface findings). All medical and surgical treatments were also recorded. Approximately a half of patients were White British but there were significant numbers of patients from other ethnic groups, South Asian and Black in particular. Oral antibiotics were the causative agent in almost a half of the patients with SJS, systemic infections in 14%, non-steroidal anti-inflammatory drugs in 8% and anticonvulsants in 7%. The age of onset was varied but a significant proportion of patients developed acute SJS in childhood. There was a significant correlation between visual acuity at initial referral to final recorded vision. Vision was found to continue to significantly deteriorate over time despite therapeutic interventions. Our regression model shows that ~62% of the variance in final vision can be explained by the initial vision and duration disease. The majority of our patients were on advanced ocular surface treatments including serum drops, topical ciclosporin and retinoic acid drops. Of particular significance, approximately a third of our patient cohort was also on systemic immune suppression. In conclusion, chronic SJS within the UK population under tertiary care remains an area of unmet clinical need. Current medical and surgical modalities prevent worsening of vision in severe ocular disease from SJS.

## Introduction

Stevens-Johnson syndrome is an immune mediated mucocutaneous disease that induces widespread sloughing of the skin and mucosal surfaces, and mortality can be as high as 35% (1). The estimated annual incidence of SJS is 1.2–12.35 new cases per million (2). SJS is a type IV hypersensitivity reaction and leads to a characteristic vesiculo-bullous reaction. Whilst the pathogenesis is unclear, it appears to be mediated by a cell-mediated keratinocyte apoptosis via the Fas signaling cascade (3). In survivors of the disease, the unremitting chronic inflammation, dessication and scarring lead to blindness, with 20–79% of survivors experiencing chronic ocular surface disease (4–6).

There are numerous potential causes for the development of SJS. The commonest is an idiosyncratic reaction to systemic medications, such as antibiotics, anti-epileptic medications and non-steroidal drugs. In 15% of cases, no causative drug agent can be found (1, 7). However, other causes include viral infections and vaccines (8). Ethnicity also plays a role and it is estimated that Asian patients are at a 2-fold increased risk of SJS compared to Caucasian patients (9).

To our knowledge, there is a lack of published literature on SJS in the United Kingdom. One study by Radford et al. in collaboration with the British Ophthalmic Surveillance Unit in 2012, reported 16 cases of SJS/TEN across the United Kingdom (10). They identified the incidence to range between 0.1 and 1.2 per million, with the highest in the West Midlands, fitting with a higher Indian sub-continent ethnicity. Greater London had an incidence of 0.2 per million. Fifty nine percent (59%) of patients had visual acuities < 6/18 at presentation which fell to 55% on their final 12-month follow-up.

The aim of the work described in this manuscript is to provide a comprehensive review of the clinical characteristics in chronic SJS patients within the United Kingdom population, their causative factors, treatment profile and prognosis. While some papers have reported cicatricial conjunctivitis as whole, we aim to provide a spotlight on ocular SJS and its sequalae. We believe this paper has the highest number of cases of ocular SJS reported in the United Kingdom, shedding some light on this rare condition.

## Methods

### Patients

This retrospective clinical was conducted at Moorfields Eye Hospital in London, UK. The study adhered to the tenets of the Declaration of Helsinki and was approved by our institution's review board (IRAS 119170). All patients referred to Moorfields Eye Hospital (London, UK) for the management of their chronic SJS related ocular surface disease were selected using our medical archives diagnosis code and were included in the study.

### Evaluation Method of Clinical Data

A thorough chart review was conducted. Demographic details were collected and included age at disease onset, ethnicity (as self-identified by the patient and following national census categories), age at presentation and precipitating agent of SJS (if known). Clinical findings included laterality and Snellen best corrected visual acuity (BCVA) at initial referral visit and at last clinical visit follow-up. The presence of lid abnormalities (e.g., entropion, distichiasis/trichiasis, and symblepharon), and ocular surface findings (e.g., dry eyes, persistent epithelial defect, keratinization and limbal stem cell deficiency) were noted. These were documented as either present or absent throughout the follow-up period. For example, the presence of any keratin on the lid margin, conjunctiva or cornea was noted as a positive finding. Limbal stem cell deficiency was defined as corneal conjunctivalization in presence of late fluorescein staining. No grading system of clinical findings was conducted given the heterogeneity of the reviewed notes and the retrospective nature of this study. All medical treatments (e.g., topical drops, systemic medications and contact lens use) and surgical treatments (e.g., lid surgery, amniotic membrane transplantation, and limbal stem cell transplantation) were also recorded. Supplementary Material 1 provides all the data collected. Given that acute care of SJS was carried out at several burn units nationally at different location sites, details of early ocular care were not available for review.

### Statistical Analysis of Clinical Data

Statistical analysis was conducted using SPSS version 24 (IBM Corp, Armonk, New York). To negate the effect of inter-eye correlation, data from only from one eye per patient was used for statistical analysis. This can be achieved through many methods, but we used the left eye for the purpose of this analysis (11, 12). Correlations were measured using the data collected, with particular focus on the association with chronic complications i.e., dry eye, symblepharon and LCSD. The strength of association between continuous variables (e.g., initial and final visual acuity) was determined using a Pearson correlation (r). The strength of association between binary and continuous variables (e.g., final visual acuity and presence of symblepharon) was determined using a point-biserial correlation. The strength of association between binary variables (e.g., presence of trichiasis and corneal graft) was determined using a Phi coefficient. An association (x) was considered strong when x ≥ |0.6|, moderate when |0.3| **≤** x <|0.6 | and weak when x < |0.3|. The Bonferroni correction was not applied due to its inconclusive value and to minimize Type II error in the study of this rare disease (13, 14). To calculate which variables could independently predict the final VA, a stepwise linear regression was performed. A *p*-value of <0.05 was considered to be statistically significant.

## Results

### Demographics

**Figure 1:**
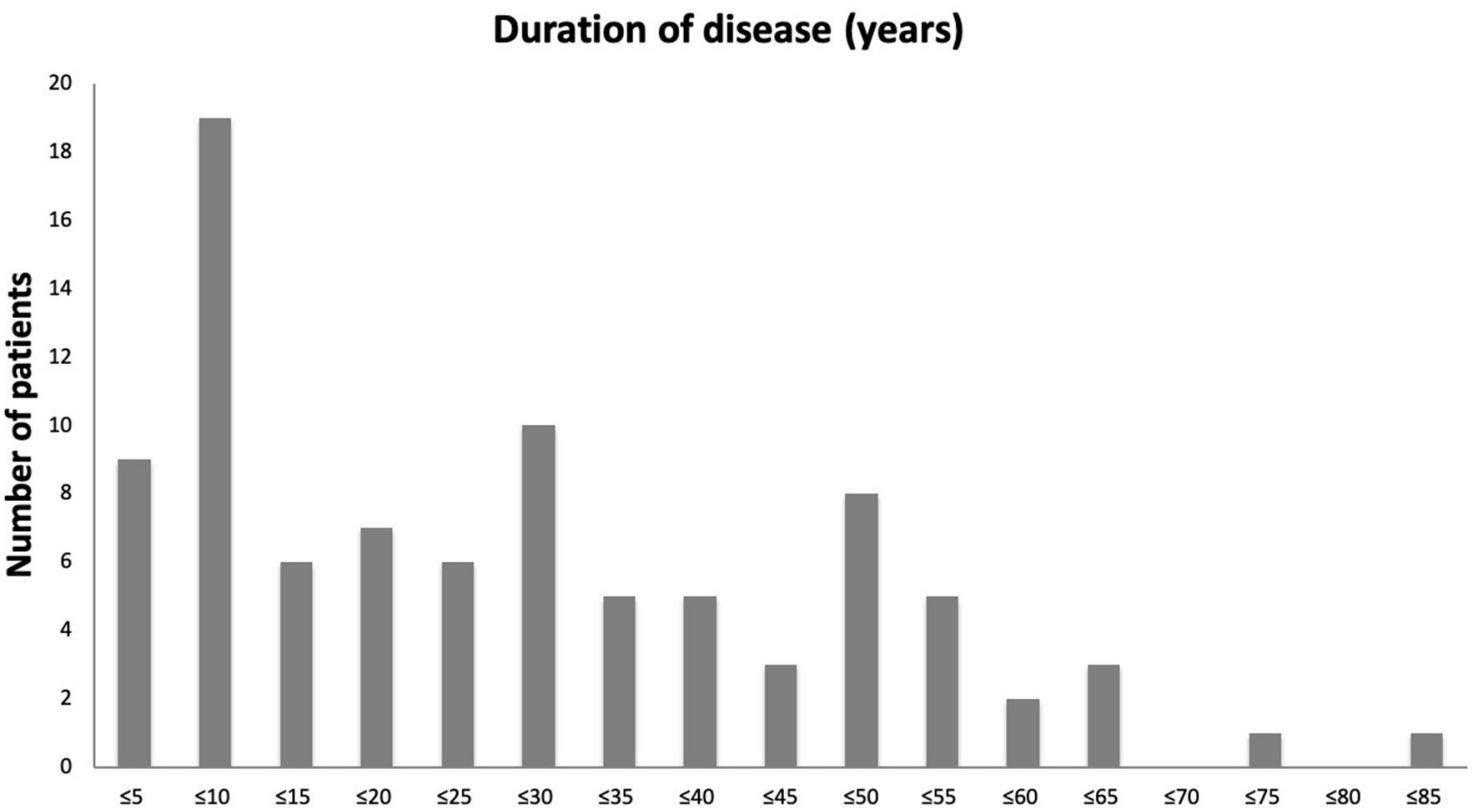
A bar chart showing age of patients at onset of acute SJS. Number of patients in every 5-year age range from ≤5 years to ≤85 years are shown.

**Figure 2:**
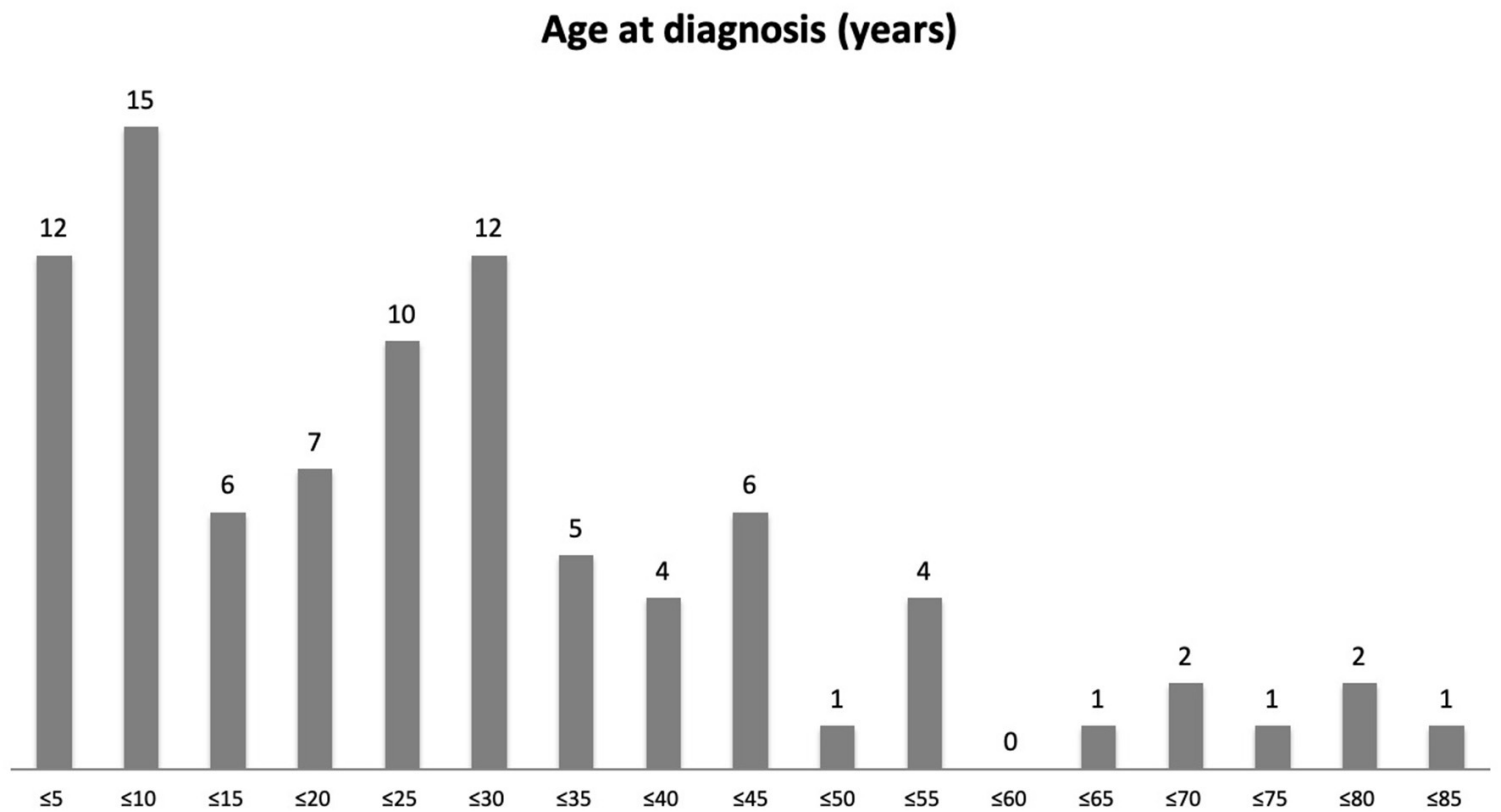
A bar graph showing duration of ocular SJS in years. Number of patients in each 5-year range category from ≤5 years to ≤85 years are shown.

**Figure 3:**
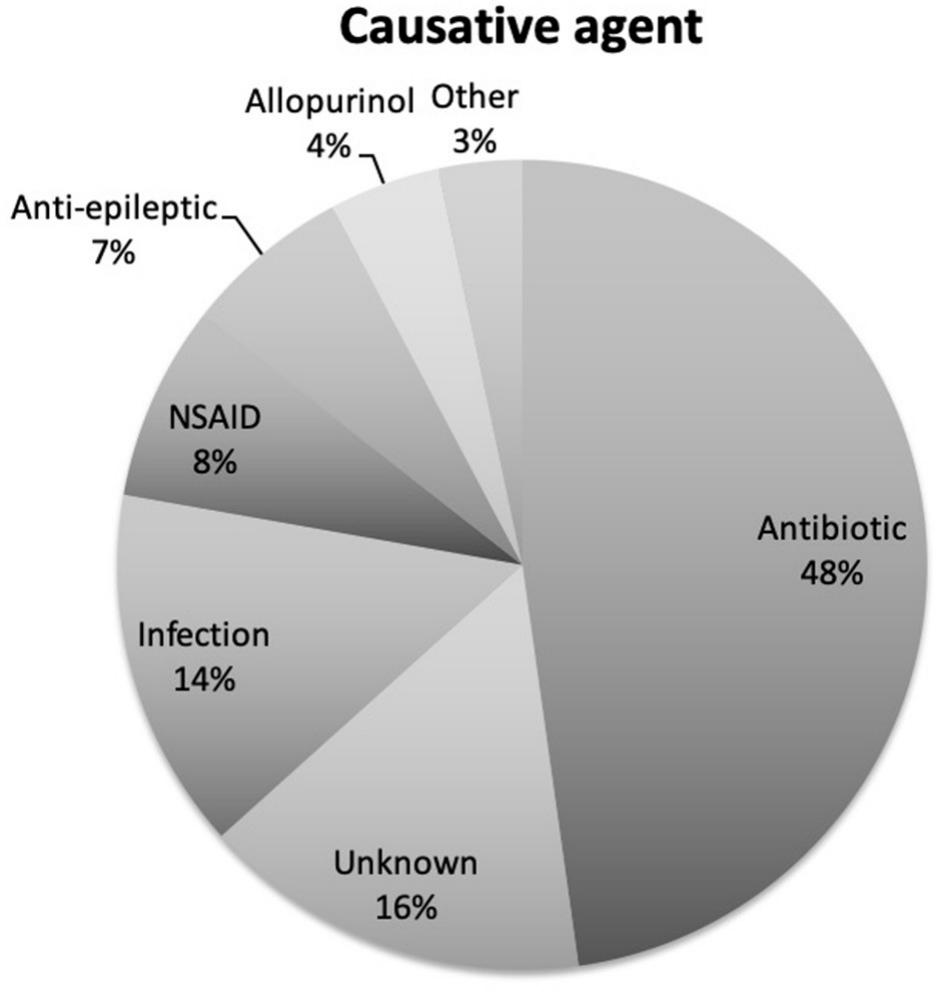
A pie chart showing the causative agent for SJS in our patient cohort. Antibiotics are the commonest cause being in almost half of our patients.

**Figure 4:**
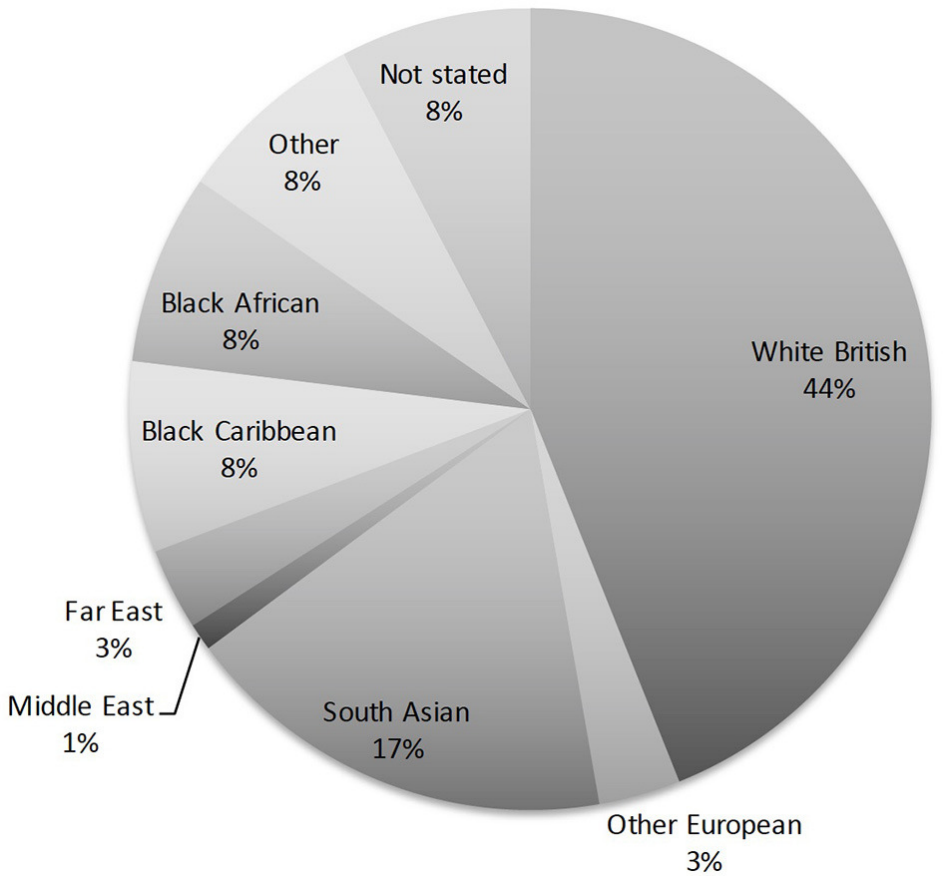
A pie chart showing the breakdown of ethnicities amongst our patients. Although most of our patients were White British (44%), over a half belonged to various other ethnic groups.

There was an almost equal gender split in our patient cohort (47 female and 44 male). The mean age of acute SJS onset was 25.4 years with a range of 3 months to 82 years (Figure 1). Table 1 provides the demographics details of our patient population. There are two peaks of disease onset, those younger than 10 years of age and those between 21 and 30 years of age. Our patients had suffered from SJS for a mean of 26.7 years (defined from the time of acute disease to last encounter) with a range of 0–81 years (Figure 2). The causative agents in almost half of our patients were antibiotics (43 patients; 48%) including penicillins, sulfa drugs and tetracyclines (Figure 3). Other common causes were systemic infections (13 patients; 14%), non-steroidal anti-inflammatory drugs (7 patients; 8%), anti-epileptics (6 patients; 7%) and allopurinol (4 patients; 4%). Almost half of our patients were White British (40 patients; 44%) but the other half was represented by different ethnicities, mostly South Asian (16 patients; 17%) and Black African or Black Caribbean (14 patients; 16%) patients (Figure 4).

**Table 1:** Patients' demographics and Snellen decimal best-corrected visual acuity.

	**Mean (range)**	**Standard Deviation**
Age at disease onset (years)	24.9 (0.0–82.0)	19.9
Age at presentation to our center (years)	52.0 (8.0–90.0)	18.4
Duration of disease (time from onset to last follow-up) (years)	0.49 (0.00–1.50)	19.44
BCVA at presentation (decimal)	0.49 (0.00–1.50)	0.39
BCVA at last follow-up (decimal)	0.44 (0.00–1.50)	0.47

*BCVA, best-corrected visual acuity*.

### Vision

**Figure 5:**
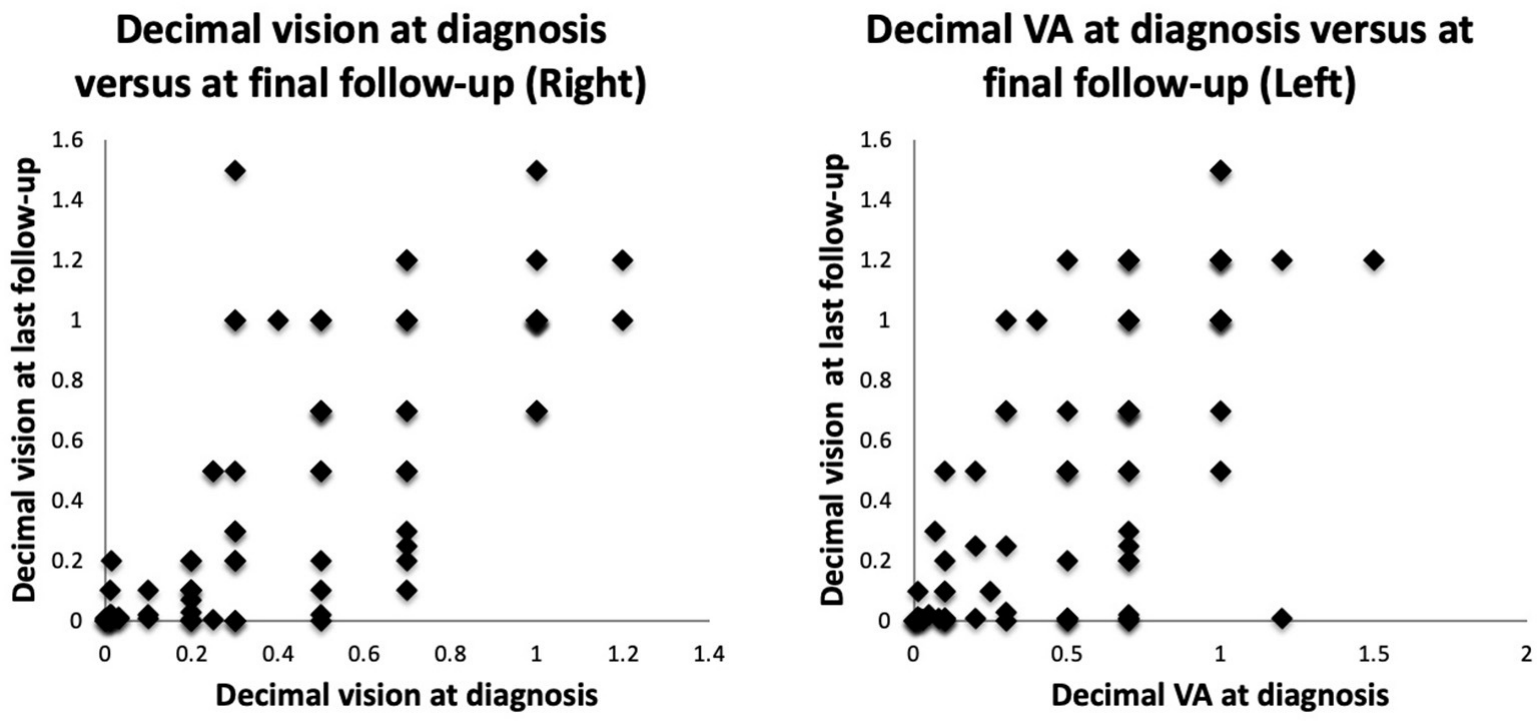
Dot plots showing decimal vision in the right eye (RE) and the left eye (LE) at presentation against most recent vision.

**Figure 6:**
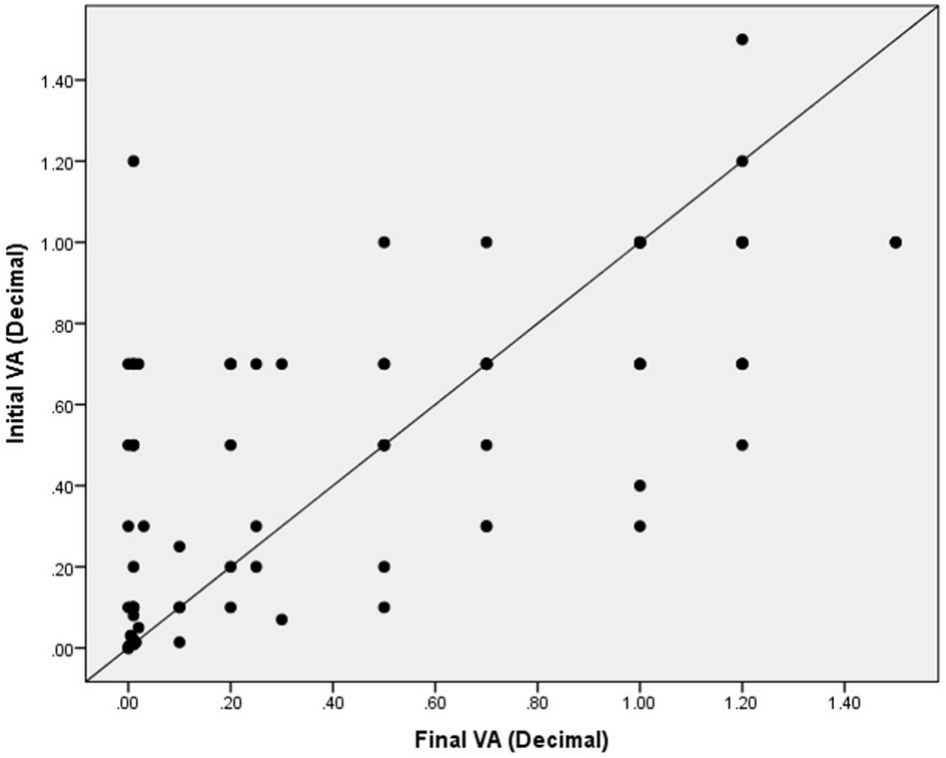
Scatter plot demonstrating the relationship between initial visual acuity and final visual acuity, as compared to a plot with a slope =1. Most eyes demonstrate a deteriorating vision throughout the follow-up.

When each eye is evaluated separately, the majority had decimal vision worse than 0.33 (6/18 or worse) (Figure 5). The initial visual acuities between the right eye and left eye on presentation were correlated (*p* = 0.001), and there was no statistically significant difference in the initial vision between each eyes (*p* = 0.754). The final visual acuities between the right eye and left eye were also correlated (*p* = 0.001) and there was no statistically significant difference in the final vision between each eye (*p* = 0.975). For both right and left eyes, poor vision remained poor at most recent follow-up despite clinical input and interventions. Vision continued to deteriorate for most eyes despite treatment. This is demonstrated in the scatter plot between initial BCVA and final BCVA (Figure 6).

### Correlation of Signs

There was evidence of moderate correlation between the presence of symblepharon and misdirected lashes (*p* = 0.001, Phi coefficient), and a weak correlation with dry eye (*p* = 0.008, Phi coefficient). There was a moderate correlation between the presence of limbal stem cell deficiency and misdirected lashes (*p* = 0.003, Phi coefficient) and weak correlation with the formation of lid margin keratin (*p* = 0.012, Phi coefficient), conjunctival keratin (*p* = 0.015, Phi coefficient) and corneal keratin (*p* = 0.008), Phi coefficient. There was a moderate correlation between the presence of corneal keratin and conjunctival keratin (*p* = 0.001, Phi coefficient), and weak correlations with the formation of lid margin keratin (*p* = 0.015, Phi coefficient) and limbal stem cell deficiency (*p* = 0.008, Phi coefficient). Supplementary Material 2 provides the table of all correlations evaluated in this study.

### Treatment

All medical and surgical interventions are presented in Table 2. Of the therapies used in our cohort of patients, 82.2% were using steroid drops, 41.1% were on Ciclosporin A drops, 22.2% were using serum drops (either autologous or allogeneic serum) and 32.2% were on topical retinoic acid. A third of our patient cohort were prescribed systemic immune suppression (mostly Mycophenolate). In patients who have used RGP or scleral lenses, final decimal visual acuity was 0.21 ± 0.10 (0.00 to 1.20) compared to 0.01 ± 0.00 (0.00 to 1.20) with those with no RGP or scleral lenses (*p* = 0.001). In patients who have undergone mucous membrane grafting (MMG) for lid keratinization, final decimal visual acuity was 0.31 ± 0.58 (0.00 to 1.50) compared to 0.56 ± 0.57 (0.01 to 1.20) at first visit (*p* = 0.236).

**Table 2:** Percentage of patients undergoing specific medical and surgical ocular interventions.

	**Percentage of patients (%)**
Topical steroids	82.2
Topical ciclosporin A	41.1
Topical serum drops (autologous or allogeneic)	22.2
Topical retinoic acid	32.2
Contact lens wear (RGP or SCL)	49.5
Amniotic membrane transplantation	18.7
Punctal closure	25.3
Systemic immunosuppression	33.3
Lid surgery (entropion, trichiasis)	80.2
Mucous membrane grafting	10.0
Corneal transplantation (penetrating or lamellar keratoplasty, keratoprosthesis)	13.2
Limbal stem cell transplantation	5.5

*RGP, rigid-gas permeable; SCL, scleral contact lens*.

### Signs Associated With Poor Vision

Our regression model predicted variance in final vision [*F*_(4, 78)_ = 31.93, *p* < 0.001, R^2^ = 0.62]. Parameters of significance included initial vision (*B* = 0.697, *p* < 0.001), corneal keratin (*B* = −0.239, *p* = 0.004), duration of disease (*B* = −0.004, *p* = 0.019) and limbal stem cell deficiency (*B* = −0.176, *p* = 0.024). There was no evidence of auto-correlation (Durbin-Watson <2.5) or multicollinearity (VIF <10), and the assumption of homoscedasticity was met. This model shows that ~62% of the variance in final vision can be explained by the initial vision, corneal keratin, duration disease and presence of limbal stem cell deficiency.

## Discussion

In this manuscript we describe our findings from the retrospective analysis of our large cohort of patients in the chronic ocular phase of SJS. The chronic ocular features of SJS include limbal stem cell deficiency and ocular surface keratinisation which lead to blindness. Our cohort is of importance partly because of the large number of patients with this rare disease but also because it shows a varied ethnic (and therefore likely genetic) spectrum with approximately a half being White British but significant numbers of South Asian and Black patients. London has a diverse ethnic mix owing to its cosmopolitan global city status and hence provides a closer representation of global epidemiological patterns. Unlike some other published cohorts, the commonest trigger in our patients was antibiotics rather than anticonvulsants (15), allopurinol (16), or non-steroidal anti-inflammatory drugs (17), although they were also represented in the trigger data. As in other published cohorts, over one third of our patients (33 patients) developed SJS in childhood (18).

Long-term visual impairment is a major problem in chronic SJS, and this is reflected in our cohort. In one case series of 89 patients, Jongkhajornpong et al. found 26% of their patient cohort developed severe visual impairment during both the acute and chronic phases of the disease, and this is also seen in other published reports (19–21). However, when further subgroup analysis was performed and chronic ocular complications were focused upon, the rate of severe visual impairment rose to 52.27%. As in our cohort, it was found that corneal neovascularisation, symblepharon and corneal opacification were the key determinants of severe visual impairment. In our model the major predictors of final VA were the initial VA duration and LCSD and keratin. As a result of these chronic complications, management of the acute stage is critical. In a recent study by Basu et al. almost 66% of children with SJS/TEN who did not receive appropriate care in the acute phase were blind 1 year after the acute episode of SJS/TEN (22). Hence, immediate acute ophthalmic assessment and intervention, and appropriate follow-up by corneal specialists is critical to prevent these significant vision compromising sequelae.

Long-term visual rehabilitation remains an area of unmet need. Very few interventions demonstrate an improvement in final visual acuity in patients with chronic SJS. The wear of scleral contact lenses, mainly the PROSE (Prosthetic Replacement of the Ocular Surface Ecosystem) lens has been shown to improve final visual acuity, similar to our outcomes with scleral or RGP lenses (22–25). Mucous membrane grafting, a newer procedure, targets lid margin keratinization. It has been shown in some studies to improve overall visual outcomes in SJS (23). Ten eyes in our cohort underwent MMG, but there was no statistically significant change in vision. More studies are warranted on the proper technique and timing of MMG in halting progressive keratinization and deterioration of vision.

This is an exploratory study of a rare disease. Correlations were ascertained to explore the relationship between a range of clinical signs with advanced sequelae of the disease. This does not explain a causal relationship nor any potential confounding between these variables. However, it does provide valuable information on potential associations that could help guide further research and management of SJS. The initial vision at a tertiary referral center does not reflect the initial vision at the time of disease onset. While this may introduce an element of Berkson bias, the strength of this study is real-world outcome data that reflects referral pathways from secondary to tertiary referral centers.

At Moorfields Eye Hospital, for all patients with severe ocular surface disease (SJS, mucous membrane pemphigoid and limbal stem cell deficiency), our management protocol is based on a treatment stepladder approach. This includes management of dryness, epithelial defects, and limbal stem cell deficiency; lid disease (trichiasis, lid malposition and meibomian gland dysfunction); and inflammation. Treatments will include the use of topical treatments (lubricants, mucolytics, preservative free steroids, ciclosporin drops, and serum drops); lid surgery (excision of lash follicles, lid split and anterior lamellar repositioning, mucous membrane grafts); the use of scleral contact lenses; amniotic membrane transplantation and surface reconstructive surgery; and systemic immune suppression.

One third of the patients in our cohort were prescribed systemic immunosuppression. This is a significant number and proportion of patients. The use of systemic immunosuppression in chronic SJS remains a controversial and poorly studied topic. In most cases with SJS, late surface failure has been associated with the destruction of corneal limbal stem cells during the acute inflammatory phase of the disease. Chronic cicatricial sequelae, such as trichiasis, entropion and exposure also contribute to progressive surface failure. Abating the acute inflammatory response with systemic immunosuppression, including corticosteroids, and addressing cicatricial lid disease can prevent and halt surface failure in patients. In a small subset of patients, disease progression can still occur despite appropriate treatment, believed to be due to endogenous recurrent conjunctival inflammation (26). A recent retrospective study described seven tertiary referrals of SJS with chronic inflammation that required systemic immunosuppression including steroids and steroid-sparing agents. Five out of these seven patients demonstrated improved outcome with therapy (27). Although specific outcomes and indications of the use of systemic immunosuppression in SJS is beyond the objectives and scope of this retrospective study, further studies are currently being conducted in our center examining outcomes in this subgroup of patients.

Compared with other international studies, our study remains one of the largest to date to record on chronic SJS findings with the Australia study reporting three cases, 95 cases in India and 70 cases in a Chinese study (28–30). In the Australian study, the incidence of SJS was reported to be 0.1 per million. Patients with SJS had a younger mean age of 43 +/−10 years (range 28–53 years). The diagnosis of SJS was found to be shorter, with a mean duration of 12.8 days (range 2–28 days), which was in keeping with the UK study (10, 28). The predominant signs seen in the Indian study was 75% of SJS patients had a BCVA of <6/18, with predominant signs being symblepharon formation, fornix shortening, ocular surface keratinisation with limbitis and corneal scarring. This contrasts with the Indian study where 57% of patients were between 21 and 40 years old (29). The duration from initial onset to time of presentation ranged from 6 days to 18 years. Intakes of drugs was the principle cause with sulfonamides being cited as the most frequent cause of SJS. Thirty three percent (33%) of patients has VA better than 6/12. The major complications cited in the Indian study were: corneal superficial punctate epitheliopathy, scarring and vascularization. Conjunctival xerosis and lid oedema were also commonly cited signs (29). By contrast, the Chinese study describes a mean age of 43.4 age, with 64% diagnosed in males, 36% in females. The commonest cause offending drugs were antibiotics (29.5%) and anticonvulsants (24.1%). Carbamazepine, allopurinol, and penicillins were the most common single offending drugs (17.5, 9.6, and 7.2%, respectively) (30).

Our study presents several limitations. First, all patients are referred to our center following acute care at different hospitals. The unique aspect of Moorfields Eye Hospital as a tertiary national center for ophthalmic care is that it receives complex SJS/TEN referrals across the country. Many of the patients are examined and managed by dermatologists or burns units according to local guidelines, and only those with ocular involvement resistant to first line treatment are referred for specialist opinion (1). As a result, we were unable to capture the acute ocular complications and management of the disease. This limitation is relevant to demonstrate differences in long term outcomes of SJS that received specific treatments such as AMT on the lid margin and ocular surface during the acute phase of the disease, which has been shown to improve outcomes and clinical prognosis. Second, we considered a wide range follow-up periods which can limit different time point of measurement in visual acuity and chronic complications. Third, given the heterogeneity of documentation of clinical findings, it was not possible to grade clinical findings as per chronic SJS grading system. Such data would have allowed us to stratify overall outcomes depending on severity of the disease and the presence and severity of specific clinical findings. Fourth, visual acuity as a metric of disease in chronic SJS and severe ocular disease is limited as many patients are unable to cooperate due to severe photophobia. Other visual metrics, such as visual function test surveys (NEI VFQ-25 or OSDI) or contrast sensitivity would portray a clearer image of visual outcomes in these patients. Finally, our case series presents only data from one large tertiary center and cannot represent the whole situation in the UK directly.

In conclusion, chronic SJS remain a poorly studied inflammatory disease in which prognostic factors are yet to be identified to guide better management. Our findings suggest that visual acuity at presentation is an important prognostic sign for long-term visual acuity, regardless of management and clinical course. Visual acuity continues to deteriorate with time in most cases. Early intervention could be mandated in some patients to prevent disease progression, particularly in patients with recurrent and chronic inflammation. The care of patients with chronic SJS remains an area of unmet clinical needs in which better algorithm based treatment modalities are needed.

## Data Availability Statement

The raw data supporting the conclusions of this article will be made available by the authors, without undue reservation.

## Ethics Statement

The studies involving human participants were reviewed and approved by IRAS. The ethics committee waived the requirement of written informed consent for participation.

## Author Contributions

All authors listed have made a substantial, direct and intellectual contribution to the work, and approved it for publication.

## Conflict of Interest

The authors declare that the research was conducted in the absence of any commercial or financial relationships that could be construed as a potential conflict of interest.
