# Supplementary figures and images for: Clinical Characteristics of Patients With Chronic Stevens-Johnson Syndrome Treated at a Major Tertiary Eye Hospital Within the United Kingdom
Source: Front Med (Lausanne). 2021 May 24;8:644795. doi: 10.3389/fmed.2021.644795 (PMC8180599; doi:10.3389/fmed.2021.644795)

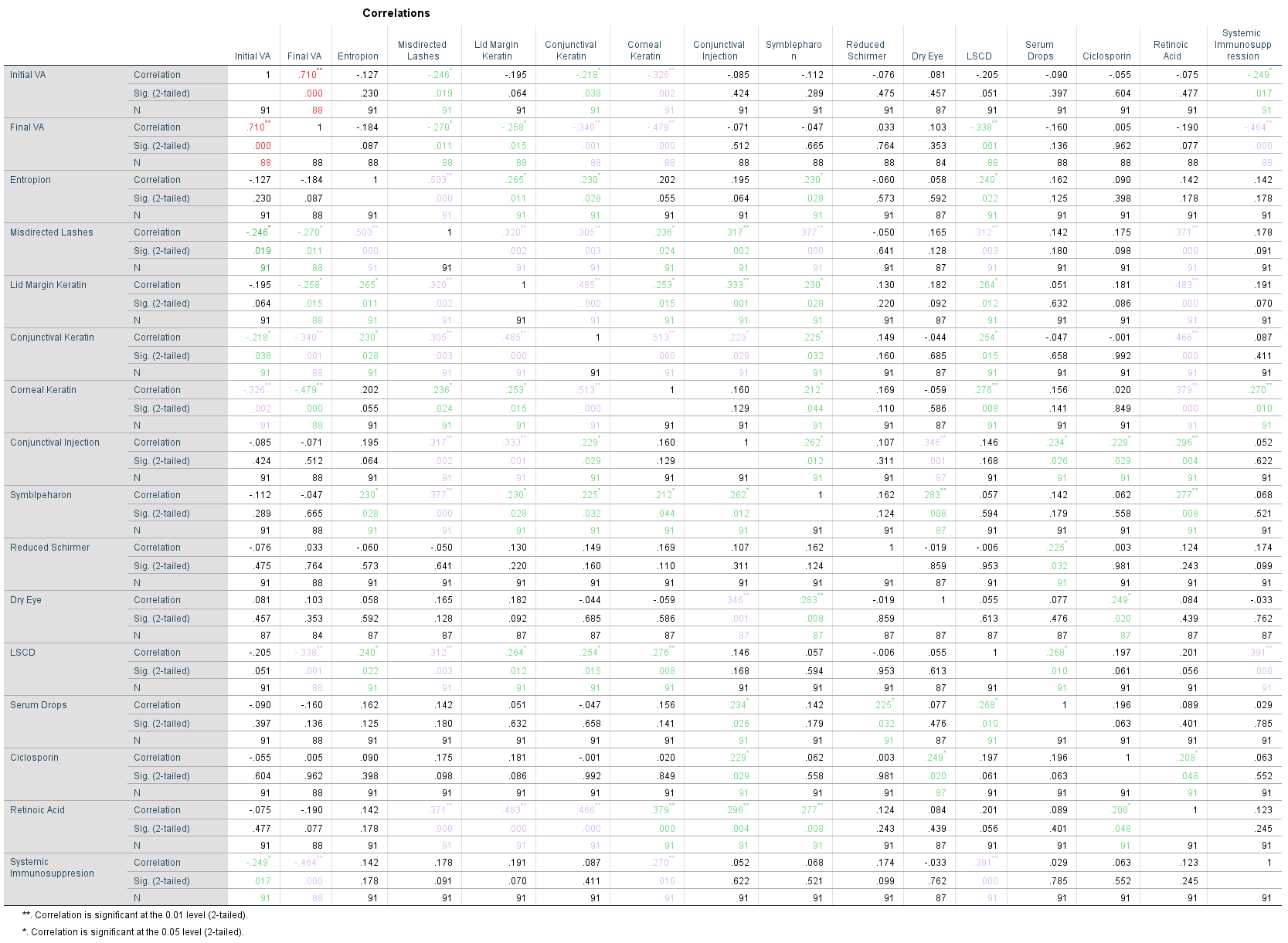

Supplement: Supplementary file 2 [file Table_2.docx]
